# Autophagy induces transforming growth factor‐β‐dependent epithelial‐mesenchymal transition in hepatocarcinoma cells through cAMP response element binding signalling

**DOI:** 10.1111/jcmm.13825

**Published:** 2018-08-22

**Authors:** Shaobo Hu, Liyu Wang, Xi Zhang, Yongzhong Wu, Jing Yang, Jun Li

**Affiliations:** ^1^ Department of Hepatobiliary Surgery Union Hospital Tongji Medical College Huazhong University of Science and Technology Wuhan China; ^2^ Department of Hepatobiliary Oncological Surgery Chongqing University Cancer Hospital Chongqing Cancer Institute Chongqing Cancer Hospital Chongqing China; ^3^ Department of Radiotherapy Chongqing University Cancer Hospital Chongqing Cancer Institute Chongqing Cancer Hospital Chongqing China; ^4^ Department of the First General Surgery Gansu Provincial Hospital Lanzhou China; ^5^ Department of Urology Oncological Surgery Chongqing University Cancer Hospital Chongqing Cancer Institute Chongqing Cancer Hospital Chongqing China

**Keywords:** autophagy, CREB signalling, epithelial‐mesenchymal transition, hepatocarcinoma cell, phosphodiesterase 4A, transforming growth factor‐β1

## Abstract

Autophagy promotes invasion of hepatocarcinoma cells through transforming growth factor (TGF)‐β‐dependent epithelial‐mesenchymal transition (EMT). This study investigated the mechanism by which autophagy induces TGF‐β‐triggered EMT and invasion of hepatocarcinoma cells. Autophagy was induced in HepG2 and BEL7402 cells by starvation in Hank's balanced salt solution. Induction of autophagy degraded phosphodiesterase (PDE) 4A and increased intracellular cAMP, PKA activity and PKA phosphorylation, resulting in increased cAMP response element binding (CREB) phosphorylation in hepatocarcinoma cells. Autophagy‐induced activation of cAMP/PKA/CREB signalling further enhanced TGF‐β1 expression, downregulated the expression of epithelial markers and upregulated the expression of mesenchymal markers, accelerating invasion of hepatocarcinoma cells. Inhibition of autophagy by Atg3 and Atg7 knockdown or by chloroquine treatment prevented degradation of PDE4A and activation of cAMP/PKA/CREB signalling, suppressing TGF‐β1 expression, EMT and invasion in hepatocarcinoma cells. In addition, inhibition of cAMP/PKA/CREB signalling also blocked autophagy‐induced TGF‐β1 expression and prevented EMT and invasion of hepatocarcinoma cells under starvation. Furthermore, exogenous inhibition of PDE4A or activation of cAMP/PKA/CREB signalling rescued TGF‐β1 expression, EMT and invasion in autophagy‐deficient hepatocarcinoma cells. These findings suggest that autophagy induces TGF‐β1 expression and EMT in hepatocarcinoma cells via cAMP/PKA/CREB signalling, which is activated by autophagy‐dependent PDE4A degradation.

## 1 INTRODUCTION

Invasion and metastasis of hepatocarcinoma cells are the leading cause of refractory cancer and poor prognosis in the clinic. Preventing invasion of hepatocarcinoma cells is an important therapeutic strategy to improve patient prognosis.^1^, ^2^ Deficiencies in angiogenesis frequently result in starvation and/or hypoxia in solid tumour cells during tumour growth, which may induce autophagy of these cells.^3^, ^4^ Autophagy has been shown to promote invasion of cancer cells during starvation or hypoxia.^5^, ^6^ We reported that autophagy accelerated invasion of hepatocarcinoma cells by inducing epithelial‐mesenchymal transition (EMT), which changed the cell phenotype from epithelial into mesenchymal under starvation conditions.^7^ Transforming growth factor (TGF)‐β is a key cytokine that induces EMT in many types of epithelial cells.^8^, ^9^ In our previous study, we showed that TGF‐β1 expression and activation of TGF‐β signalling played crucial roles in autophagy‐induced EMT and invasion of hepatocarcinoma cells.^7^ However, the mechanism underlying autophagic induction of TGF‐β in EMT and invasion of hepatocarcinoma cells is unclear.

cAMP response element binding protein (CREB) is a phosphorylation‐dependent transcription factor that is phosphorylated by multiple protein kinases and participates in different protein kinase signal transduction pathways.^10^ Activation of the CREB signalling pathway and phosphorylation of CREB were shown to fulfill numerous cellular functions ranging from cell proliferation and the cell cycle to cell differentiation and cytokine production by binding of phosphorylated‐CREB to the cAMP response element (CRE) in target genes and promoting their transcription.^11^, ^12^, ^13^, ^14^ A CRE site has been identified in the TGF‐β gene promoter,^15^, ^16^, ^17^ and p‐CREB induces TGF‐β expression in both normal cells and cancer cells by directly binding to the TGF‐β gene promoter, which contributes to tissue fibrosis or tumour progression.^15^, ^18^

The cAMP/PKA cascade is a classical signalling transduction pathway that phosphorylates CREB and regulates target gene expression. Binding of cAMP to regulatory subunits of PKA releases its catalytic subunits and phosphorylates downstream target molecules. This signalling pathway is regulated by adenylate cyclase and phosphodiesterase (PDE), which controls synthesis and hydrolysis of cAMP.^19^ Aberrant activation or inhibition of the cAMP/PKA/CREB pathway has been confirmed to result in cellular dysfunction and participates in tumour progression.^20^, ^21^

Autophagy is a lysosome‐dependent protein and organelle degradation mechanism that maintains the homoeostasis of the cellular metabolic pool. This process also modulates multiple cellular signalling pathways and physiological functions by degrading redundant proteins or enzymes under stress, such as starvation or hypoxia.^22^ Thus, this study investigated the role of autophagy in PDE4 degradation and cAMP/PKA/CREB signalling modulation as a mechanism to induce TGF‐β‐triggered EMT and invasion of hepatocarcinoma cells under starvation.

## 2 MATERIALS AND METHODS

### 2.1 Cell culture and treatment

The human hepatocarcinoma cell line HepG2 was purchased from ATCC (Manassas, VA, USA). The BEL7402 cell line was obtained from the Institute of Cell Biology, Chinese Academy of Sciences (Shanghai, China). Both cell lines were kept in the laboratory of General Surgery, Union Hospital, Tongji Medical College, Huazhong University of Science and Technology and were authenticated on December 2017. Cell lines were cultured in RPMI 1640 medium supplemented with 10% fetal bovine serum (HyClone, Logan, UT, USA) and 100 μg/mL each of penicillin and streptomycin (Gibco, Invitrogen, Carlsbad, CA, USA) in 5% CO_2_ at 37°C. Cells at passages 3‐8 were used for the experiments.

HepG2 and BEL7402 cells were, respectively, cultured in the above complete medium and starved in Hank's balanced salt solution (HBSS; HyClone) for 0, 6, 12 and 24 hours to test the PDE4A degradation kinetics in the absence or presence of autophagy. For inhibition of autophagy, cells were transfected with siRNA‐Atg3 and siRNA‐Atg7 (as described below) or treated with chloroquine (5 μmol/L; Sigma‐Aldrich, Shanghai, China) in HBSS and complete medium for 24 hours. Treatment of HepG2 and BEL7402 cells with H 89 2HCl (30 μmol/L; Selleck, Shanghai, China) or SB431542 (10 μmol/L; Selleck) in HBSS and in complete medium for 24 hours was used to specifically inhibit PKA or the TGF‐β receptor, respectively. Cell lines transfected with siRNA‐control or siRNA‐Atgs (3 and 7) were cultured in complete medium and in HBSS with roflumilast (20 nmol/L; Selleck) or 8‐bromo‐cAMP (100 μmol/L; Selleck) for 24 hours to evaluate the necessity of cAMP/PKA/CREB signalling in autophagy‐induced TGF‐β expression and EMT. The cells cultured in complete medium without treatment served as a control.

### 2.2 siRNA synthesis and transfection

Synthesis and transfection of siRNAs was performed as previously described.^7^ Briefly, the cDNA sequences of the Atg3 and Atg7 genes were obtained from GenBank (NM_022488 and NM_006395), and the targeting sequences of three different siRNA were designed using an RNAi algorithm available online https://rnaidesigner.thermofisher.com/rnaiexpress/. All siRNAs were synthesized and purified by GenePharma (Shanghai, China). The siRNA (sense: 5′‐GGGAAAGGCACUGGAAGUG‐3′, antisense: 5′‐CACUUCCAGUGCCUUUCCC‐3′) with the greatest silencing effect of Atg3 and the siRNA (sense: 5′‐ACUAAAAGGGGCAAACUGCAG‐3′, antisense: 5′‐GCAGUUUGCCCCUUUUAGUAG‐3′) with the greatest silencing effect of Atg7 were identified by real‐time PCR, and another siRNA (sense: 5′‐UCAGACAUGCAACGUCAGCU‐3′, antisense: 5′‐AGCCUUACGGGAAUCGAAUA‐3′) served as the siRNA‐vector control. These synthesized siRNAs were transfected into HepG2 and BEL7402 cells using TransLipid Transfection Reagent (Beijing, China) according to the manufacturer's instruction. After 48 hours of transfection, cell viability was evaluated by trypan blue, and cells were then treated and/or starved as described above for the subsequent experiments.

### 2.3 cAMP assay

Intracellular cAMP concentrations of HepG2 and BEL7402 cells that were treated as described above were measured using a cAMP ELISA kit (Cayman Chemical, Ann Arbor, MI, USA). Briefly, 1 × 10^5^ cells were lysed in 0.1 M HCl for 20 minutes and then were scraped off and dissociated until the suspension was homogeneous. After centrifugation at 1000 *g* for 10 minutes, the cAMP concentration of each supernatant was measured according to the manufacturer's instruction. Briefly, 50 μL of each supernatant was added to 50 μL of cAMP AChE Tracer and 50 μL of cAMP ELISA antiserum in each well. After incubation at 4°C for 18 hours, the wells were rinsed, and 200 μL per well of Ellman's reagent was added. After incubation in the dark for 2 hours, the absorbance was measured at OD = 420 nm. The cAMP concentration of each sample was calculated according to the standard curve.

### 2.4 PKA activity measurement

Intracellular PKA kinase activity of HepG2 and BEL7402 cells with the above treatments was measured using a PKA kinase activity assay kit from Abcam (ab139435; Cambridge, MA, USA) according to the manufacturer's instruction. In brief, cells were lysed in lysis buffer for 10 minutes and then were scraped and centrifuged at 16 260 *g* for 15 minutes. After determination of the protein concentration, each supernatant was diluted with Kinase Dilution Assay Buffer. Then, 30 μL of each supernatant was reacted with 10 μL of reconstituted ATP in each well at 30°C for 90 minutes. After the contents were removed, 40 μL of the PKA phosphospecific substrate antibody was added in each well and incubated at room temperature for 60 minutes. After the wells were washed, 40 μL of diluted anti‐rabbit IgG‐HRP conjugate was added to each well and incubated at room temperature for 30 minutes. After another wash, 40 μL per well of TMB substrate was added and incubated at room temperature for 60 minutes. The reaction in each well was stopped by addition of 20 μL of stop solution, and the absorbance was measured at OD = 450 nm. The PKA activity of each sample was calculated according to the standard curve.

### 2.5 Quantitative RT‐PCR

Real‐time PCR was used to detect the mRNA expression levels of PDE4A in HepG2 and BEL 7402 cells cultured in complete medium and in HBSS for 6, 12 and 24 hours, as well as the mRNA expression levels of TGF‐β1 in the above cells with different treatments. In brief, total RNA from these cells was isolated by TRIzol™ Reagent (Invitrogen) according to the manufacturer's protocol. Total RNA was reverse transcribed into first strand cDNA using an iScript cDNA Synthesis kit (Bio‐Rad, München, Germany). RNA expression was analysed by RT‐PCR using iQ SYBR Green Supermix in an iCycler Real‐Time PCR Detection System (Bio‐Rad). The following primer sequences were used: PDE4A: sense 5′‐AACTTTCCGCAGACGCCTT‐3′, antisense 5′‐ TCTGAGCGGTACAGGAAGGA‐3′, TGF‐β1: sense 5′‐AACTACTGCTTCAGCTCCAC‐3′, antisense 5′‐AGGACCTTGCTGTACTGTGT‐3′.^23^ Expression was normalized to that of β‐actin.

### 2.6 Western blotting

Western blotting was used to detect the protein expression levels of PDE4A in HepG2 and BEL 7402 cells cultured in complete medium and HBSS for 6, 12 and 24 hours, as well as the protein expression levels of TGF‐β1, PKA/CREB signalling molecules and epithelial‐mesenchymal markers in the above cells with different treatments. Cells were lysed in radioimmunoprecipitation assay (RIPA) buffer supplemented with a protease inhibitor cocktail (Roche, Branford, CT, USA) and phosphatase inhibitor cocktail (Cell Signaling Technology, Beverly, MA, USA). Total protein (30 μg) from each sample was electrophoresed on 12% SDS‐PAGE gels. After being transferred to nitrocellulose membranes (Pierce, Thermo Fisher Scientific, Inc., Waltham, MA, USA), protein samples were incubated with the following primary antibodies: Atg3 (1:1000; Abcam), Atg7 (1:1000; Abcam), LC3 (1:1000; Cell Signaling Technology), p62 (1:1000; Cell Signaling Technology), PDE4A (1:1000; Abcam), PKA (1:1000; Cell Signaling Technology), p‐PKA (Thr 197) (1:1000; Cell Signaling Technology), CREB (1:1000; Cell Signaling Technology), p‐CREB (Ser133) (1:1000; Cell Signaling Technology), TGF‐β1 (1:1000; Abcam), E‐cadherin (1:1000; Abcam), Cytokeratin18 (CK18) (1:1000; Abcam), Fibronectin (1:1000; Abcam) and Vimentin (1:1000; Abcam). Blots were incubated with the appropriate horseradish peroxidase‐conjugated secondary antibodies, and the membranes were developed with SuperSignal™ chemiluminescence reagent (Pierce, Thermo Fisher Scientific, Inc.) according to the manufacturer's protocol. Protein expression levels were normalized against total‐CREB or β‐actin. Optical density of the bands was quantified using NIH (Bethesda, MD, USA) ImageJ.

### 2.7 Immunofluorescence

Expression levels of TGF‐β1 and epithelial‐mesenchymal markers in the above HepG2 and BEL 7402 cells with different treatments were detected by immunofluorescence using an avidin‐biotin peroxidase complex method. Briefly, cells were fixed in 4% paraformaldehyde and permeabilized using Triton‐X‐100. Cells were then treated in 3% hydrogen peroxide to inactivate endogenous peroxidase. Non‐specific binding was blocked in PBS containing 10% species‐appropriate normal serum for 1 hour at room temperature. The above primary antibodies for TGF‐β1 (1:100), E‐cadherin (1:200), Cytokeratin18 (1:200), Fibronectin (1:200) and Vimentin (1:200) were applied overnight at 4°C in a humidified chamber. Cells were incubated with the appropriate secondary antibody and visualized by peroxidase substrate in conjunction with FITC or Cy3 (Boster, Wuhan, China).

### 2.8 ELISA

First, 5 × 10^6^ HepG2 and BEL7402 cells that were transfected with siRNA‐Atg3 and siRNA‐Atg7 or with siRNA‐control were cultured in HBSS and complete medium for 24 hours. Then, 5 × 10^6^ HepG2 and BEL7402 cells were treated with chloroquine (5 μmol/L) or H 89 2HCl (30 μmol/L) in HBSS and complete medium for 24 hours as described above. Each supernatant was then collected to detect the TGF‐β1 concentration by enzyme‐linked immunosorbent assays (ELISAs) using ELISA kits (Abcam) according to the manufacturer's instructions.

### 2.9 Invasion assay

The invasiveness of the above HepG2 and BEL 7402 cells with different treatments was analysed using Matrigel‐coated invasion chambers containing 8‐μm pore filters (BD Biosciences, San Jose, CA, USA). Briefly, each cell line with the above treatments was seeded at a density of 1 × 10^5 ^cells per well in the upper Matrigel‐coated chamber of 24‐well plates. HBSS was placed in the upper chamber. Complete medium was placed in the lower chamber as a chemotactic agent. Cells that were seeded in the upper chamber with complete medium served as a control. Invasion assay systems were incubated at 37°C with 5% CO_2_ for 6 hours. Cells that invaded the Matrigel to the bottom of filter were stained with 2 μg/mL DAPI in PBS and counted under a fluorescence microscope.

### 2.10 Statistical analysis

All data are presented as the mean ± SEM. After demonstration of homogeneity of variance with a Bartlett test, one‐way ANOVA, followed by Student‐Newman‐Keuls test where appropriate, was used to evaluate the statistical significance. Values of *P* < 0.05 were considered statistically significant. Experiments were performed in triplicate.

## 3 RESULTS

### 3.1 Autophagy degrades PDE4A in hepatocarcinoma cells

**Figure 1:**
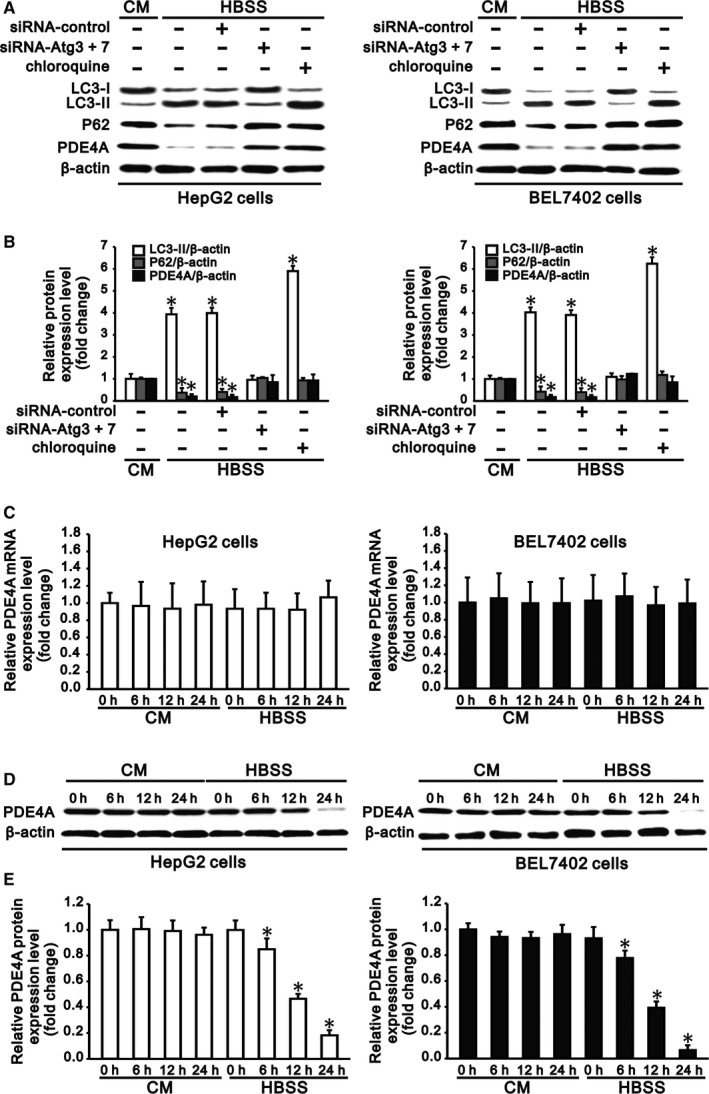
Autophagy contributes to degradation of PDE4A in hepatocarcinoma cells. Starvation of HepG2 and BEL7402 cells in Hank's balanced salt solution (HBSS) for 24 h‐induced autophagy, which was shown by the LC3‐I to LC3‐II conversion and P62 degradation. Induction of autophagy significantly reduced PDE4A expression in hepatocarcinoma cells. Treatment of cells in HBSS with chloroquine (5 μmol/L) for 24 h or combined transfection of cells with siRNA‐Atg3 and siRNA‐Atg7 inhibited autophagy and preserved expression of PDE4A in hepatocarcinoma cells. A, Representative Western blots and (B) densitometric analysis of LC3‐I, LC3‐II, P62 and PDE4A normalized to β‐actin in HepG2 (left panel) and BEL7402 cells (right panel) cultured in complete medium (CM) or in HBSS with different treatments. Cells that were cultured in CM served as a control. C, PDE4A degradation kinetics at the mRNA level in HepG2 (left panel) and BEL7402 cells (right panel) that were cultured in CM and HBSS for 0, 6, 12, and 24 h were determined by quantitative RT‐PCR. mRNA levels of PDE4A were normalized to that of β‐actin. D, Representative Western blots and (E) densitometric analysis for PDE4A degradation kinetics normalized to β‐actin in HepG2 (left panel) and BEL7402 cells (right panel) that were cultured in CM and in HBSS for 0, 6, 12, and 24 h. Cells that were cultured in CM at the initial time (0 h) served as the control. Data are representative of three independent experiments and are shown as the mean ± SEM, n = 3, *P < 0.05 vs control

HepG2 and BEL7402 cells were starved by culturing in HBSS for 6 hours. The autophagic flux was determined by Western blot analysis of LC3‐I to LC3‐II conversion and P62 degradation. In contrast to culture in complete medium, starvation of cells in HBSS upregulated LC3‐II expression and downregulated expression of LC3‐I and P62, indicating the induction of autophagy in hepatocarcinoma cells under starvation. Following the induction of autophagy, PDE4A expression, along with P62, was significantly reduced in HepG2 and BEL7402 cells (Figure 1A,B).

To further confirm the role of autophagy in degradation of PDE4A in hepatocarcinoma cells, we inhibited autophagy of cells by combined transfection of siRNA‐Atg3 and siRNA‐Atg7 or chloroquine treatment. After silencing of Atg3 and Atg7 (Figure S1), LC3‐II conversion was reduced and expression of P62 was increased in HepG2 and BEL7402 cells. On the other hand, chloroquine treatment increased the LC3‐II and P62 expression in cells, also indicating the inhibition of autophagy in hepatocarcinoma cells under starvation (Figure 1A,B). In contrast to autophagic induction, inhibition of autophagy significantly increased PDE4A expression, along with P62, in HepG2 and BEL7402 cells (Figure 1A,B). These results indicated that autophagy controlled degradation of PDE4A in hepatocarcinoma cells.

To further clarify the kinetics of PDE4A degradation in hepatocarcinoma cells under starvation by autophagy, we cultured HepG2 and BEL 7402 cells in complete medium and HBSS for 6, 12 and 24 hours. Real‐time PCR showed that the RNA level of PDE4A in cells was not changed by complete medium and HBSS or by different culture times (Figure 1C). Western blotting showed that the protein level of PDE4A in cells was also not changed by complete medium at different culture time points; however, the protein expression was reduced to 83% and 74%; 47% and 37%; and 18% and 6% in HepG2 and BEL 7402 cells after 6, 12 and 24 hours, respectively, of culture in HBSS compared with that of the initial time (Figure 1D,E). These results confirmed that PDE4A of hepatocarcinoma cells was degraded by autophagy at the post‐transcriptional level.

### 3.2 Autophagy promotes activation of cAMP/PKA/CREB signalling in hepatocarcinoma cells

**Figure 2:**
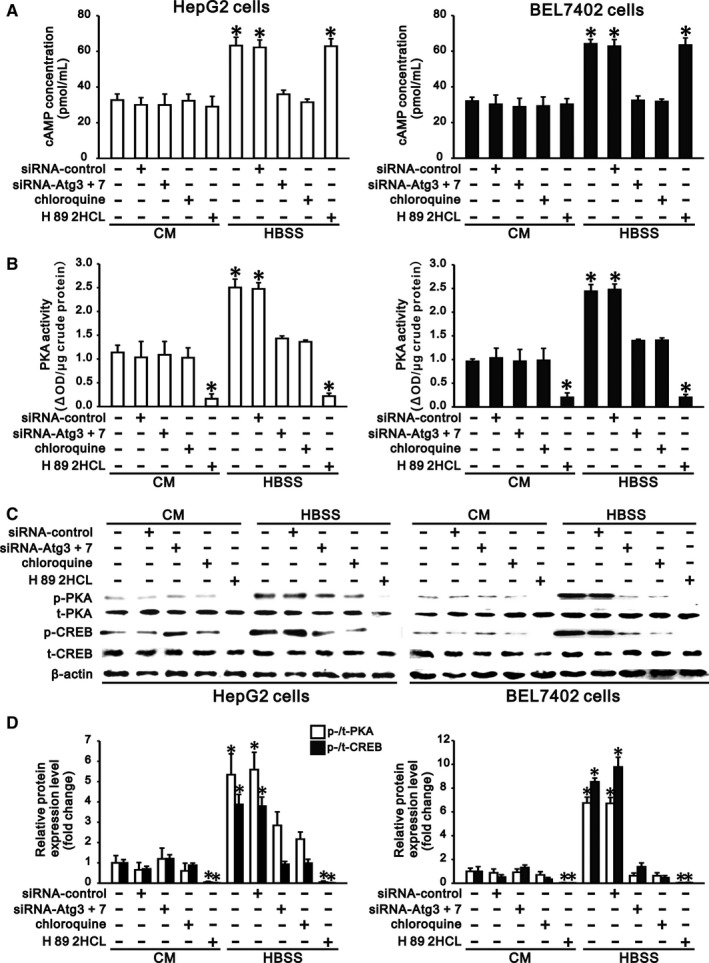
Autophagy promotes activation of cAMP/PKA/cAMP response element binding (CREB) signalling in hepatocarcinoma cells. HepG2 and BEL 7402 cells transfected with siRNA‐control or siRNA‐Atgs (3 and 7) were incubated in complete medium (CM) and Hank's balanced salt solution (HBSS) for 24 h, and the cells without transfection were treated with chloroquine (5 μmol/L) or H 89 2HCl (30 μmol/L) in CM and HBSS for 24 h. Induction of autophagy in HBSS increased intracellular cAMP concentration (A) and PKA activity (B), as well as phosphorylation of PKA and CREB (C,D), in HepG2 (left panel) and BEL7402 cells (right panel). Inhibition of autophagy by Atg3/7 knockdown or by chloroquine (5 μmol/L) treatment reduced intracellular cAMP concentration (A) and PKA activity, (B) as well as phosphorylation of PKA and CREB (C,D), in hepatocarcinoma cell lines in HBSS compared with cells in CM. Treatment of cells with H 89 2HCl (30 μmol/L), a specific PKA inhibitor, significantly decreased intracellular PKA activity and inhibited PKA and CREB phosphorylation in hepatocarcinoma cell lines both in CM and in HBSS. C, Representative Western blots and (D) densitometric analysis for phosphorylated (p)‐PKA normalized to total (t)‐PKA and phosphorylated (p)‐CREB normalized to total (t)‐CREB. Cells that were cultured in CM without treatment served as the control. Data are representative of 3 independent experiments and are shown as the mean ± SEM, n = 3, *P < 0.05 vs control

PDE4 negatively regulates the cAMP/PKA/CREB signalling pathway by hydrolysing cAMP. Since PDE4A in HepG2 and BEL7402 cells was degraded by starvation in HBSS (Figure 1), starvation of these cells in HBSS significantly increased the intracellular cAMP concentration, PKA activity and PKA phosphorylation compared with those in complete medium (Figure 2). Phosphorylation of CREB, a downstream effector molecule, in hepatocarcinoma cells was also significantly increased following activation of PKA under starvation (Figure 2C,D). However, inhibition of autophagy by Atg knockdown or chloroquine treatment significantly reduced the starvation‐induced increase in intracellular cAMP concentration, PKA activity and PKA phosphorylation, resulting in decreased CREB phosphorylation in hepatocarcinoma cells under starvation compared to cells in complete medium (Figure 2). To further confirm the role of cAMP/PKA signalling in the autophagy‐induced increase in CREB phosphorylation, we treated HepG2 and BEL7402 cells with H 89 2HCl, a specific inhibitor of PKA, in both complete medium and HBSS. We found that H 89 2HCl prevented both basal and starvation‐induced activation of PKA in hepatocarcinoma cells, resulting in abrogated basal and starvation‐induced phosphorylation of CREB (Figure 2). These results demonstrated that autophagy promotes activation of cAMP/PKA/CREB signalling in hepatocarcinoma cells under starvation.

### 3.3 cAMP/PKA/CREB signalling contributes to autophagy‐induced TGF‐β1 expression in hepatocarcinoma cells

p‐CREB was reported to induce TGF‐β1 expression by binding to the CRE site in the TGF‐β1 gene promoter.^15^ Since activation of cAMP/PKA/CREB signalling was significantly promoted by autophagy (Figure 2), and autophagy also induced TGF‐β1 expression in hepatocarcinoma cells under starvation,^7^ we further investigated the role of cAMP/PKA/CREB signalling in autophagy‐induced TGF‐β1 expression in HepG2 and BEL7402 cells.

**Figure 3:**
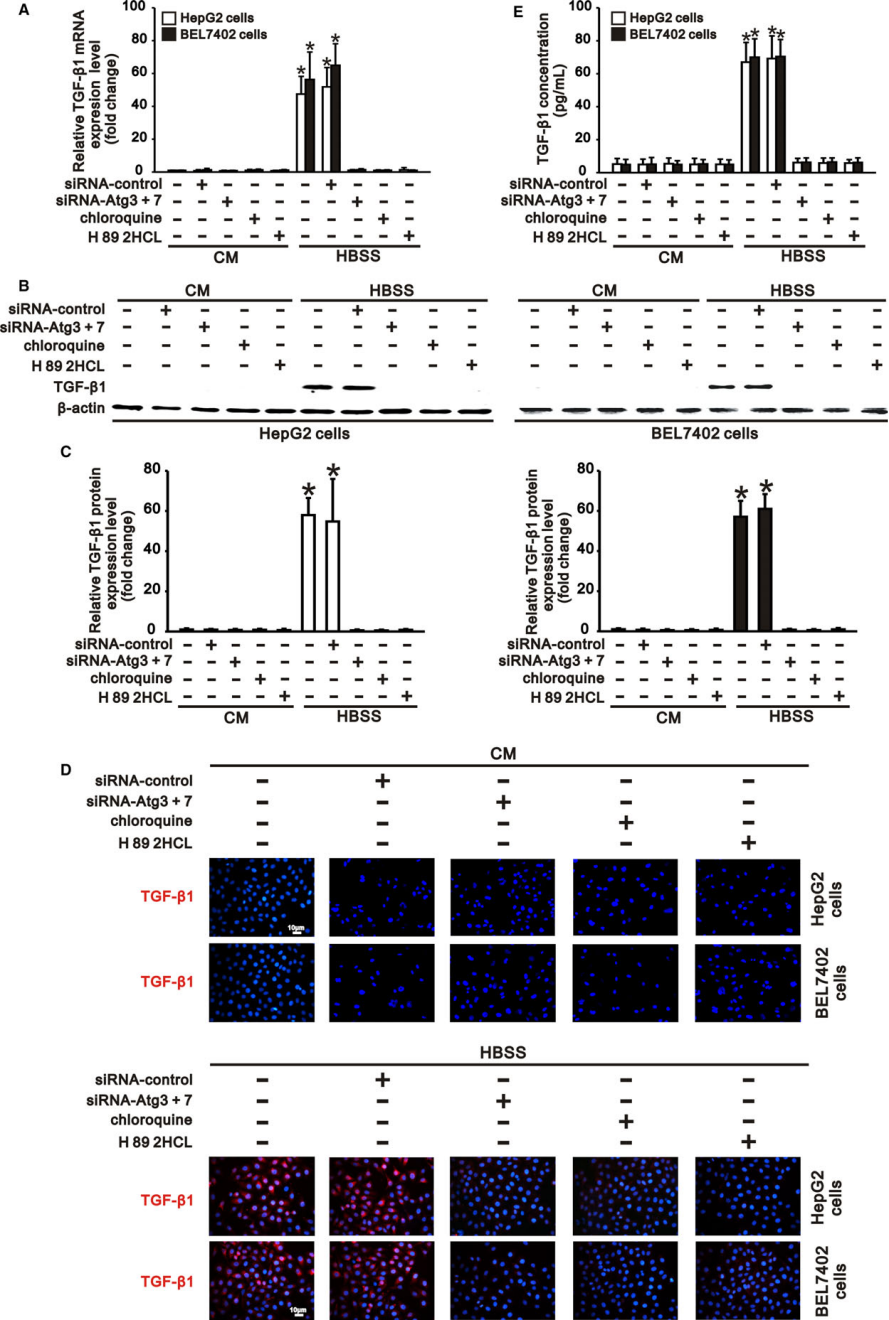
Autophagy induces transforming growth factor (TGF)‐β1 expression via cAMP/PKA/cAMP response element binding protein (CREB) signalling in hepatocarcinoma cells. HepG2 and BEL 7402 cells transfected with siRNA‐control or siRNA‐Atgs (3 and 7) were incubated in complete medium (CM) and HBSS for 24 h, and the cells without transfection were treated with chloroquine (5 μmol/L) or H 89 2HCl (30 μmol/L) in complete medium and HBSS for 24 h. Expression of TGF‐β1 in hepatocarcinoma cells was induced by autophagy induction in HBSS and was reduced by autophagy inhibition through Atg3/7 knockdown or chloroquine (5 μmol/L) treatment compared to that of cells in CM. Inhibiting PKA activation by H 89 2HCl (30 μmol/L) significantly downregulated autophagy‐induced TGF‐β1 expression in hepatocarcinoma cells under starvation. A, Expression of mRNA for TGF‐β1 in HepG2 and BEL7402 cells was determined by quantitative RT‐PCR. mRNA levels were normalized to that of β‐actin. B, Representative Western blots and (C) densitometric analysis for TGF‐β1 normalized to β‐actin in HepG2 (left panel) and BEL7402 cells (right panel). D, Representative immunofluorescence staining for TGF‐β1 in HepG2 and BEL 7402 cells (scale bar: 10 μm, magnification ×200). E, Concentrations (pg/mL) of TGF‐β1 in the above CM or HBSS after 24 h incubation were measured by ELISA. Cells cultured in CM without treatment served as the control. Data are representative images or are expressed as the mean ± SEM (n = 3) from 3 separate experiments. *P < 0.05 vs control

We found that HepG2 and BEL7402 cells in complete medium did not express TGF‐β1 at both the RNA and protein levels, corresponding to the low basal activation of cAMP/PKA/CREB signalling (Figures 2 and 3). Compared with the cells in complete medium, starvation of these cells in HBSS significantly induced TGF‐β1 expression and increased the TGF‐β1 concentration in HBSS, consistent with the increased activation of cAMP/PKA/CREB signalling (Figures 2 and 3). However, inhibition of autophagy by Atg knockdown or chloroquine treatment, which inhibited activation of cAMP/PKA/CREB signalling, significantly reduced starvation‐induced TGF‐β1 expression in hepatocarcinoma cells (Figures 2 and 3). Furthermore, treatment of cells in HBSS with H 89 2HCl, which inhibited PKA/CREB signalling activation (Figure 2), also notably reduced autophagy‐induced TGF‐β1 expression at both the RNA and protein levels (Figure 3). These results indicated that autophagy‐induced TGF‐β1 expression in a cAMP/PKA/CREB signalling‐dependent manner in hepatocarcinoma cells.

### 3.4 cAMP/PKA/CREB signalling mediates autophagy‐induced EMT and invasion of hepatocarcinoma cells

**Figure 4:**
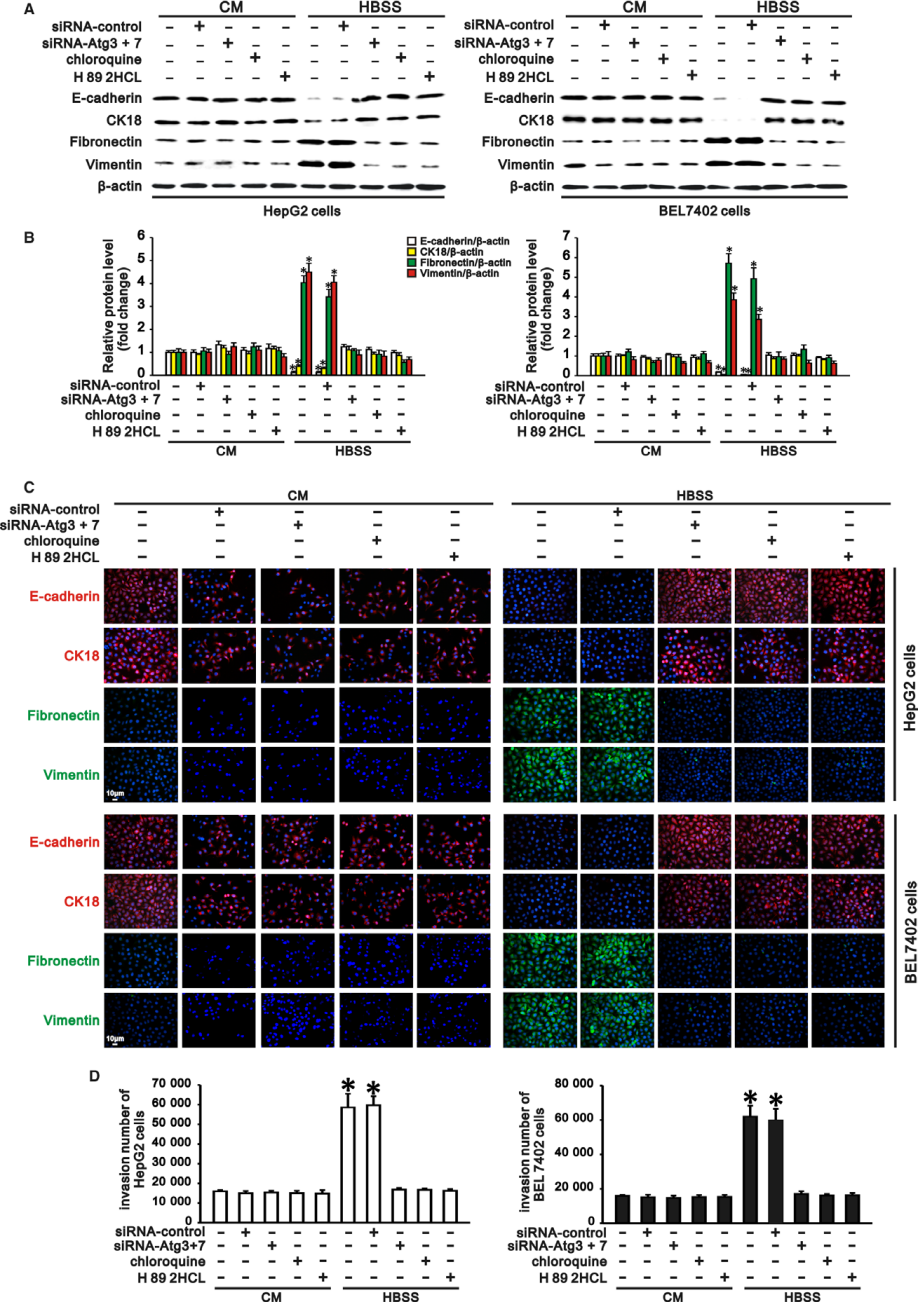
cAMP/PKA/cAMP response element binding protein (CREB) signalling contributes to autophagy‐induced epithelial‐mesenchymal transition (EMT) and invasion of hepatocarcinoma cells. HepG2 and BEL 7402 cells transfected with siRNA‐control or siRNA‐Atgs (3 and 7) were incubated in complete medium (CM) and Hank's balanced salt solution (HBSS) for 24 h, and the cells without transfection were treated with chloroquine (5 μmol/L) or H 89 2HCl (30 μmol/L) in CM and HBSS for 24 h. Induction of autophagy in HBSS‐induced mesenchymal marker expression and suppressed epithelial marker expression (EMT), along with increased invasion of hepatocarcinoma cells. Inhibition of autophagy by Atg3/7 knockdown or chloroquine (5 μmol/L) treatment suppressed EMT and invasion of hepatocarcinoma cells in HBSS compared with cells in CM. Inhibition of PKA activation by H 89 2HCl (30 μmol/L) treatment abrogated autophagy‐induced EMT and invasion of hepatocarcinoma cells under starvation. A, Representative Western blots and (B) densitometric analysis for E‐cadherin, CK18 (epithelial markers), fibronectin and vimentin (mesenchymal markers) normalized to β‐actin in HepG2 (left panel) and BEL7402 cells (right panel). C, Representative immunofluorescence staining for E‐cadherin, CK18, fibronectin and vimentin in HepG2 (upper panel) and BEL 7402 cells (lower panel) (scale bar: 10 μm, magnification ×200). D, Invasive numbers of HepG2 (left panel) and BEL7402 cells (right panel) in CM and HBSS with different treatments. Cells cultured in complete medium without treatment served as a control. Data are representative images or are expressed as the mean ± SEM (n = 3) from 3 separate experiments. *P < 0.05 vs control

We previously found that autophagy promoted invasion of hepatocarcinoma cells through TGF‐β‐dependent EMT^7^ and that cAMP/PKA/CREB signalling contributed to autophagy‐induced TGF‐β1 expression. These findings prompted us to investigate whether cAMP/PKA/CREB signalling played roles in EMT and invasion of hepatocarcinoma cells with autophagy. Western blotting and immunostaining revealed that HepG2 and BEL7402 cells in complete medium expressed high levels of E‐cadherin and CK18 (epithelial markers) but low levels of fibronectin and vimentin (mesenchymal markers). Starvation of these cells in HBSS significantly downregulated the E‐cadherin and CK18 levels and upregulated the fibronectin and vimentin levels (Figure 4A‐C). However, inhibition of autophagy by Atg knockdown or chloroquine treatment upregulated starvation‐reduced expression of E‐cadherin and CK18 but downregulated starvation‐induced expression of fibronectin and vimentin in hepatocarcinoma cells compared with cells in complete medium. More importantly, treatment of HepG2 and BEL7402 cells with H 89 2HCl in HBSS enhanced epithelial marker expression and reduced mesenchymal marker expression (Figure 4A‐C), indicating that cAMP/PKA/CREB signalling inhibition could abrogate autophagy‐induced EMT in hepatocarcinoma cells. Moreover, as a characteristic of EMT in cancer cells, the invasiveness of hepatocarcinoma cells was also evaluated in this study. In accordance with the above changes in EMT marker expression, starvation of HepG2 and BEL7402 cells in HBSS significantly enhanced their invasiveness compared with that of cells in complete medium. Either inhibition of autophagy or inhibition of cAMP/PKA/CREB signalling significantly attenuated autophagy‐induced invasion of HepG2 and BEL7402 cells in HBSS (Figure 4D). Thus, cAMP/PKA/CREB signalling plays a crucial role in autophagy‐induced EMT and invasion of hepatocarcinoma cells.

### 3.5 Autophagy‐induced EMT and invasion of hepatocarcinoma cells is dependent on autophagy‐induced TGF‐β1

**Figure 5:**
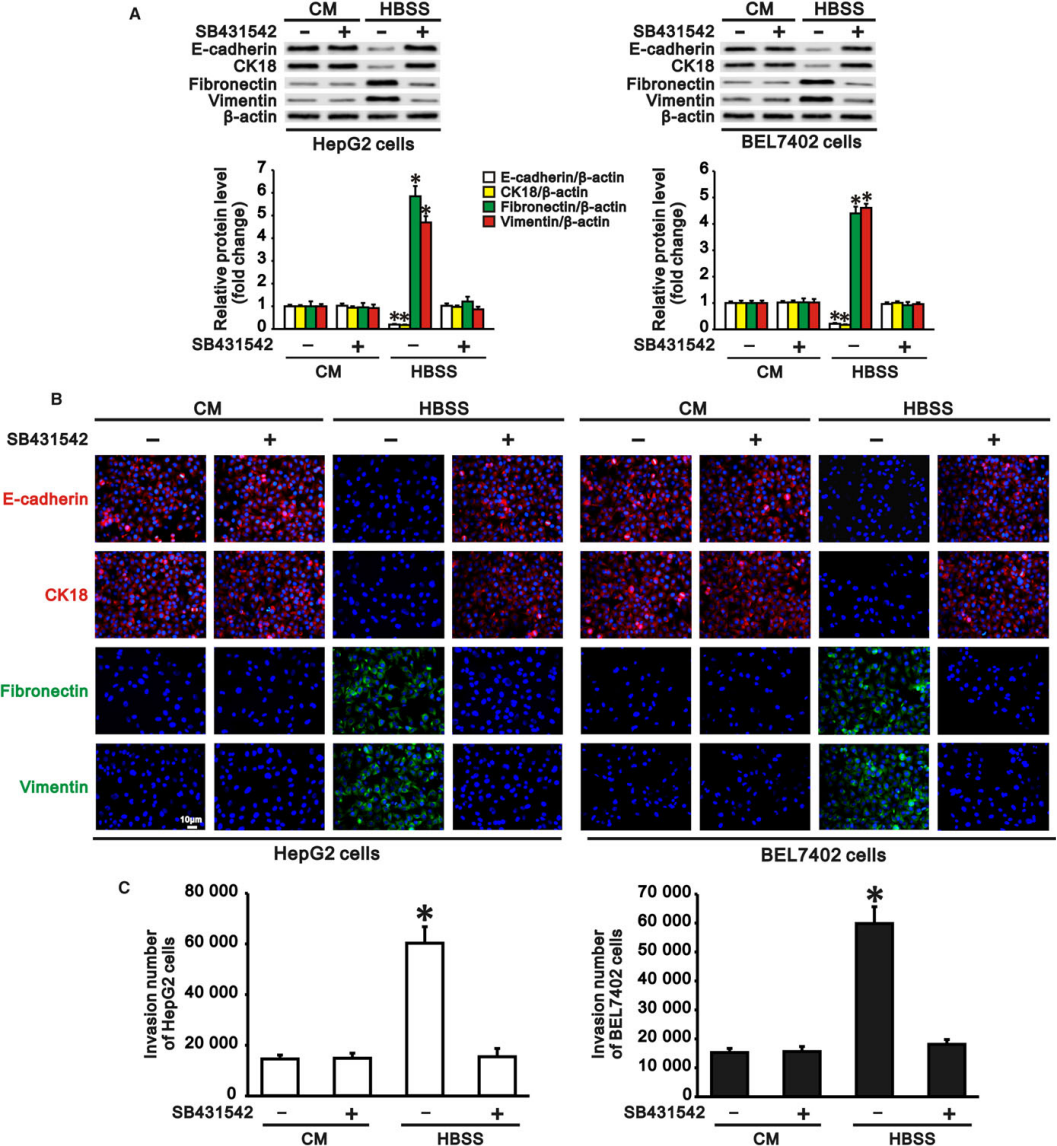
Blockade of autophagy‐induced transforming growth factor (TGF)‐β1 interferes with autophagy‐induced epithelial‐mesenchymal transition (EMT) and invasion of hepatocarcinoma cells. HepG2 and BEL7402 cells were treated with SB431542 (10 μmol/L), a TGF‐β receptor inhibitor, in complete medium (CM) and Hank's balanced salt solution (HBSS) for 24 h. Treatment with SB431542 inhibited the autophagy‐induced EMT and invasion of hepatocarcinoma cells during starvation compared with complete medium. A, Representative Western blots (upper panel) and densitometric analysis (lower panel) for E‐cadherin, CK18 (epithelial markers), fibronectin and vimentin (mesenchymal markers) normalized to β‐actin in HepG2 and BEL7402 cells with or without SB431542 treatment in CM and HBSS. B, Representative immunofluorescence staining for E‐cadherin, CK18, fibronectin and vimentin in HepG2 (left panel) and BEL 7402 cells (right panel) with or without SB431542 treatment in CM and HBSS (scale bar: 10 μm, magnification ×200). C, Invasive numbers of HepG2 (left panel) and BEL7402 cells (right panel) with or without SB431542 treatment in CM and HBSS. Cells cultured in complete medium without treatment served as a control. Data are representative images or are expressed as the mean ± SEM (n = 3) from three separate experiments. *P < 0.05 vs control

Since cAMP/PKA/CREB signalling plays crucial roles both in autophagy‐induced TGF‐β1 expression and in autophagy‐induced EMT, and TGF‐β is a key cytokine that induces EMT in many types of epithelial cells,^8^, ^9^ we further investigated the role of the autophagy‐induced TGF‐β1 in EMT and invasion of hepatocarcinoma cells with autophagy during starvation. We treated HepG2 and BEL7402 cells with SB431542 (10 μmol/L), a TGF‐β receptor inhibitor, both in complete medium and HBSS to block the effect of TGF‐β1. We found that treatment of these cells with SB431542 inhibited the autophagy‐induced downregulation of epithelial marker expression and upregulation of mesenchymal marker expression during starvation compared with complete medium (Figure 5A,B). As expected, SB431542 also reduced the invasive number of HepG2 and BEL7402 cells during starvation when EMT was inhibited by inactivity of the TGF‐β receptor (Figure 5C).

### 3.6 Activation of cAMP/PKA/CREB signalling rescues TGF‐β1 expression, EMT and invasion in autophagy‐deficient hepatocarcinoma cells

**Figure 6:**
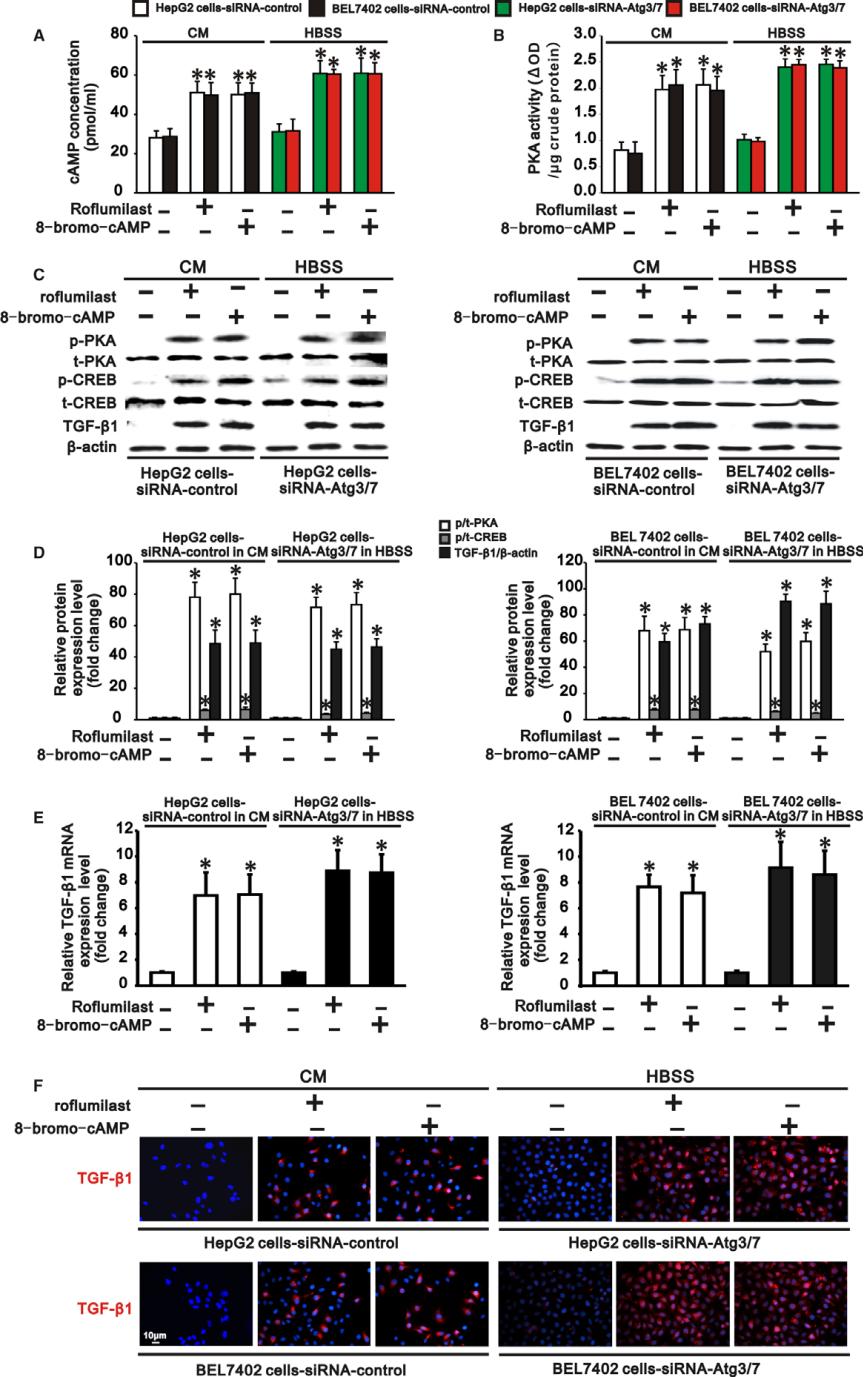
Inhibition of PDE4 or activation of cAMP/PKA/cAMP response element binding protein (CREB) signalling rescues transforming growth factor (TGF)‐β1 expression in autophagy‐deficient hepatocarcinoma cells. HepG2 and BEL 7402 cells transfected with siRNA‐control or siRNA‐Atgs (3 and 7) were, respectively, cultured in complete medium (CM) and Hank's balanced salt solution (HBSS) to induce autophagy deficiency, and these two types of autophagy‐deficient cells were then treated with a PDE4 inhibitor, roflumilast (20 nmol/L), or a PKA activator, 8‐bromo‐cAMP (100 μmol/L), for 24 h. Roflumilast or 8‐bromo‐cAMP treatment increased intracellular cAMP concentration (A) and PKA activity (B), as well as phosphorylation of PKA and CREB (C,D), which further induced TGF‐β1 (C‐F) expression in these two types of autophagy‐deficient hepatocarcinoma cells. C, Representative Western blots and (D) densitometric analysis of p‐PKA, p‐CREB and TGF‐β1, respectively, normalized to t‐PKA, t‐CREB and β‐actin in HepG2 (left panel) and BEL7402 cells (right panel) transfected with siRNA‐control or siRNA‐Atgs (3 and 7) in CM or in HBSS. E, Quantitative mRNA level of TGF‐β1 normalized to β‐actin in HepG2 (left panel) and BEL7402 cells (right panel) transfected with siRNA‐control or siRNA‐Atgs (3 and 7) in CM or in HBSS. F, Representative immunofluorescence staining for TGF‐β1 in HepG2 (upper panel) and BEL 7402 cells (lower panel) transfected with siRNA‐control or siRNA‐Atgs (3 and 7) in CM or in HBSS (scale bar: 10 μm, magnification ×200). Transfected cells without treatment served as a control. Data are representative images or are expressed as the mean ± SEM (n = 3) from three separate experiments. *P < 0.05 vs control

**Figure 7:**
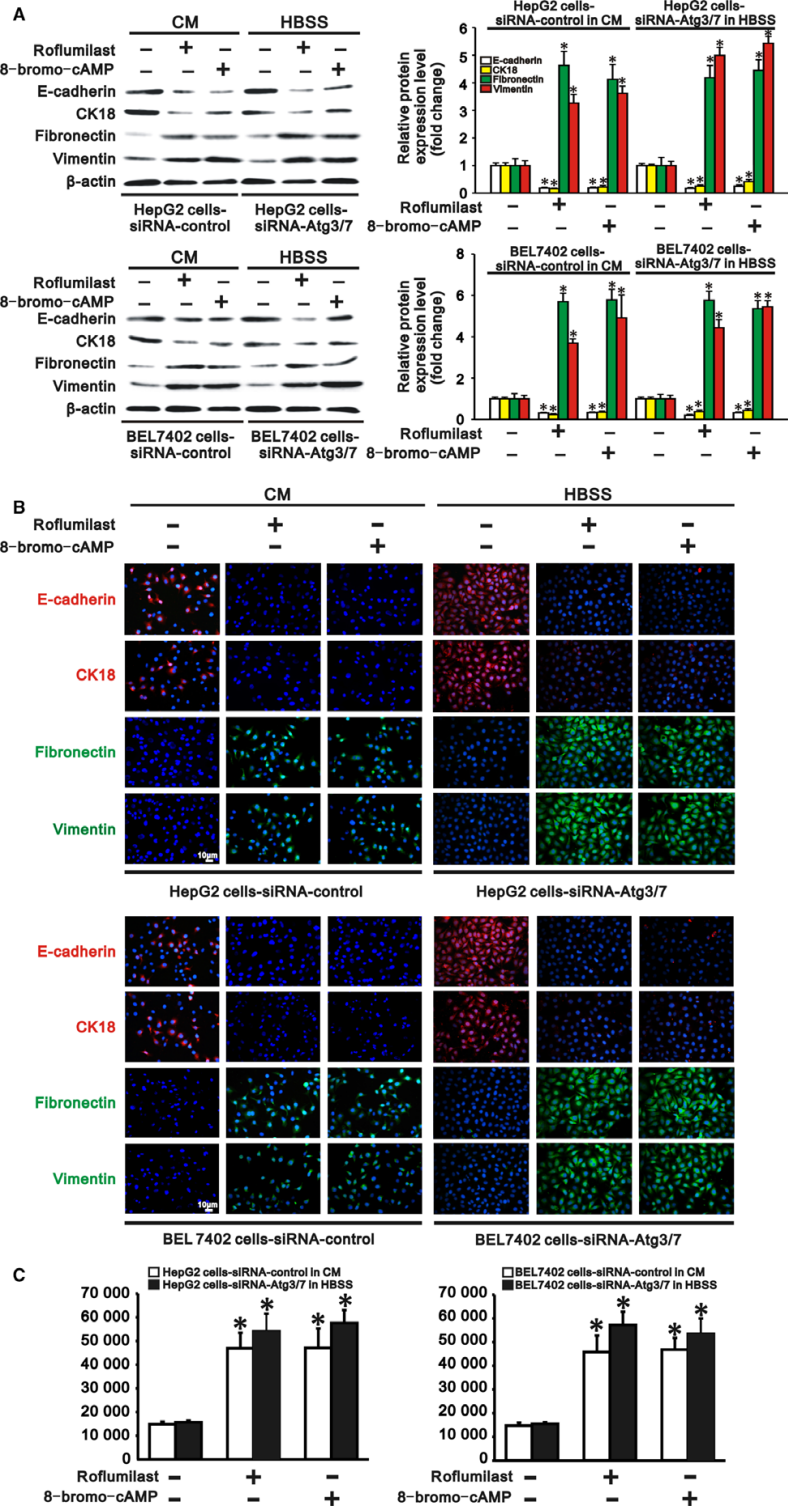
PDE4 inhibition or cAMP/PKA/cAMP response element binding (CREB) signalling activation remedies epithelial‐mesenchymal transition (EMT) and invasion of autophagy‐deficient hepatocarcinoma cells. HepG2 and BEL 7402 cells transfected with siRNA‐control or siRNA‐Atgs (3 and 7) were, respectively, cultured in complete medium (CM) and Hank's balanced salt solution (HBSS) to cause autophagy deficiency, and these two types of autophagy‐deficient cells were then treated with a PDE4 inhibitor, roflumilast (20 nmol/L), or a PKA activator, 8‐bromo‐cAMP (100 μmol/L), for 24 h. Treatment of these autophagy‐deficient hepatocarcinoma cells with roflumilast or 8‐bromo‐cAMP significantly downregulated their E‐cadherin and CK18 (epithelial markers) levels but upregulated fibronectin and vimentin (mesenchymal markers) levels and enhanced their invasiveness. A, Representative Western blots (left panel) and densitometric analysis (right panel) of E‐cadherin, CK18, fibronectin and vimentin normalized to β‐actin in HepG2 and BEL 7402 cells transfected with siRNA‐control or siRNA‐Atgs (3 and 7) in CM or in HBSS. B, Representative immunofluorescence staining for E‐cadherin, CK18, fibronectin and vimentin in HepG2 (upper panel) and BEL 7402 cells (lower panel) transfected with siRNA‐control or siRNA‐Atgs (3 and 7) in CM or in HBSS (scale bar: 10 μm, magnification ×200). C, Invasive numbers of HepG2 (left panel) and BEL7402 cells (right panel) transfected with siRNA‐control or siRNA‐Atgs (3 and 7) in CM or in HBSS. Transfected cells without treatment served as a control. Data are representative images or are expressed as the mean ± SEM (n = 3) from 3 separate experiments. *P < 0.05 vs control

Since autophagy activated cAMP/PKA/CREB signalling by degrading PDE4A in hepatocarcinoma cells, we further tested whether inhibiting PDE4A or activating cAMP/PKA/CREB signalling could rescue TGF‐β1 expression, EMT and invasion in autophagy‐deficient hepatocarcinoma cells. HepG2 and BEL 7402 cells transfected with siRNA‐control or siRNA‐Atgs were cultured in complete medium and HBSS to cause autophagy deficiency. These two types of autophagy‐deficient hepatocarcinoma cells were then treated with a PDE4 inhibitor, roflumilast (20 nmol/L), or a PKA activator, 8‐bromo‐cAMP (100 μmol/L), for 24 hours. We found that both roflumilast and 8‐bromo‐cAMP significantly increased intracellular cAMP concentration, PKA activity and PKA phosphorylation, as well as phosphorylation of CREB, in these two types of autophagy‐deficient hepatocarcinoma cells (Figure 6A‐D). These results indicated that cAMP/PKA/CREB signalling was activated by PDE4 inhibition or PKA activation in autophagy‐deficient hepatocarcinoma cells. In parallel with the finding that activation of cAMP/PKA/CREB signaling induced TGF‐β1 expression in hepatocarcinoma cells, we also found that TGF‐β1 was upregulated at both the RNA and protein levels in these autophagy‐deficient hepatocarcinoma cells by roflumilast or 8‐bromo‐cAMP treatment (Figure 6C‐F). As a consequence, expression of the epithelial markers E‐cadherin and CK18 was reduced, while expression of the mesenchymal markers fibronectin and vimentin was increased in these autophagy‐deficient hepatocarcinoma cells (Figure 7A,B), demonstrating that EMT occurred in these autophagy‐deficient hepatocarcinoma cells following PDE4 inhibition or PKA activation. Finally, the invasiveness of the autophagy‐deficient HepG2 and BEL7402 cells was also accelerated compared to that of cells without roflumilast or 8‐bromo‐cAMP treatment (Figure 7C). Collectively, activation of cAMP/PKA/CREB signalling either by PDE4 inhibition or by PKA activation rescued TGF‐β1 expression, EMT and invasion in autophagy‐deficient hepatocarcinoma cells.

## 4 DISCUSSION

Anti‐angiogenesis therapy is a common strategy that promotes cancer cell apoptosis or necrosis through nutrient deprivation or hypoxia.^3^, ^4^ However, exposure of cancer cells to starvation or hypoxia leads to cellular autophagy, which promotes cell survival by generating intracellular nutrients, growth factors and energy under stress.^24^ Autophagy was shown to promote cancer cell invasion during starvation or hypoxia.^5^, ^6^ Hence, elucidating how autophagy mediates invasion of cancer cells under stress would help improve the curative effects and reduce the side effects of anti‐tumour therapy. We previously reported that autophagy promoted invasion of hepatocarcinoma cells by inducing TGF‐β‐dependent EMT.^7^ In this study, we describe a further mechanism by which autophagy induces TGF‐β1 expression, EMT and invasion in hepatocarcinoma cells. We showed that autophagy degraded PDE4A in hepatocarcinoma cells and increased the intracellular cAMP and activation of PKA, resulting in increased phosphorylation of CREB under starvation. The activation of cAMP/PKA/CREB signalling in hepatocarcinoma cells upregulated TGF‐β1 expression, which further induced EMT and promoted invasion of hepatocarcinoma cells. Inhibition of PKA/CREB signalling downregulated autophagy‐induced TGF‐β1 expression and prevented both EMT and invasion of hepatocarcinoma cells. However, either inhibiting PDE4A or activating PKA/CREB signalling was sufficient to rescue TGF‐β1 expression, EMT and invasion in autophagy‐deficient hepatocarcinoma cells. These findings suggest that TGF‐β1‐mediated EMT and invasion of hepatocarcinoma cells induced by autophagy under starvation are dependent on PDE4A degradation‐activated cAMP/PKA/CREB signalling.

Autophagy is a lysosome‐dependent protein degradation mechanism that maintains the homoeostasis of the cellular metabolic pool by degrading redundant or misfolded proteins or damaged organelles under stress, such as starvation or hypoxia.^22^ P62 is a multi‐domain protein that is degraded by autophagy and serves as an adaptor for degrading specific substrate proteins by autophagy. The substrate protein interacts with different domains of P62 and forms an aggregate structure, which is further transported to the autophagosome through the LC3‐interacting region of P62. The autophagic aggregate cargos with substrate proteins then undergo non‐ubiquitinated or ubiquitinated degradation by autophagy.^25^ PDE4A was reported to directly bind to Phox and the Bem1p domain of P62 and form a reversible protein aggregate that links to the autophagic degradation pathway.^26^, ^27^, ^28^ In this study, we found that the expression levels of both PDE4A and P62 were significantly downregulated by autophagy, indicating the degradation of PDE4A with P62 by autophagy in hepatocarcinoma cells under starvation. Further analysis of the expression kinetics of PDE4A in HepG2 and BEL7402 cells showed that the RNA expression level of PDE4A in hepatocarcinoma cells was not changed by culturing in complete medium or starving in HBSS for 0‐24 hours. However, the protein level of PDE4A in hepatocarcinoma cells was reduced to approximately 40% after 12 hours of starvation in HBSS but was not changed in complete medium from 0 to 24 hours, further demonstrating that PDE4A was degraded at the post‐transcriptional level under starvation in HBSS. Although previous studies did not show the degradation of PDE4A by autophagy through formation of aggregates with P62 in CHO, HEK or PC12 cell lines under pharmacological treatment,^26^, ^28^ our results suggest that the degradation of PDE4A by autophagy may be cell type and stressor‐dependent.

Numerous studies have clarified the role of various signalling pathways, ranging from AMPK/mTOR to PI3K/Akt and MAPK signalling, in malignant cell behaviours by regulating cellular autophagy.^29^, ^30^, ^31^, ^32^, ^33^ However, few studies have elucidated the mechanisms of how autophagy regulates downstream signalling to mediate cellular activities in cancer. Our previous study showed that autophagy promotes invasion of hepatocarcinoma cells by inducing TGF‐β1‐dependent EMT.^7^ Here, we provided further evidence showing that autophagy‐induced TGF‐β1 expression in hepatocarcinoma cells by activating cAMP/PKA/CREB signalling, consistent with previous reports demonstrating that p‐CREB can induce TGF‐β gene expression both in normal cells and in cancer cells by directly binding to the CRE site in the TGF‐β gene promoter.^15^, ^16^, ^17^ This autophagy‐induced activation of cAMP/PKA/CREB signalling was dependent on degradation of PDE4A based on the fact that inhibition of autophagy preserved PDE4A expression but inactivated cAMP/PKA/CREB signalling; however, inhibition of PDE4A‐activated cAMP/PKA/CREB signalling in autophagy‐deficient hepatocarcinoma cells, indicating a negative regulatory mechanism of cAMP/PKA/CREB signalling triggered by autophagy in hepatocarcinoma cells under starvation. The current data also revealed that autophagy‐activated cAMP/PKA/CREB signalling induced EMT and invasion in hepatocarcinoma cells, which was consistent with the findings that CREB plays key roles in TGF‐β‐mediated EMT and invasion of cells.^34^, ^35^

We previously reported that autophagy‐induced EMT and invasion of hepatocarcinoma cells depended on TGF‐β1 expression and activation of its downstream Smad3 signalling.^34^, ^35^ Here, we showed that autophagy‐activated cAMP/PKA/CREB signalling plays a critical role in inducing TGF‐β1 expression in hepatocarcinoma cells, which finally induces EMT and invasion of hepatocarcinoma cells. Consequently, using TGF‐β1 as a pivot, we reported the upstream and downstream mechanisms of how autophagy promotes EMT and invasion of hepatocarcinoma cells. Combined with the results of our previous study, the data indicated that autophagy activates cAMP/PKA/CREB signalling in hepatocarcinoma cells by degrading PDE4A, which then induces TGF‐β1 expression, and TGF‐β1 further activates downstream Smad3 signalling, resulting in EMT and invasion of hepatocarcinoma cells. These studies suggest that autophagy induces growth factor production and mediates cellular activities, such as EMT and invasion of cells, under stress not only by providing amino acids but also by promoting intracellular initiative signalling. In addition to autophagy and TGF‐β signalling, PDE4A and the cAMP/PKA/CREB pathway were identified as promising therapeutic targets to prevent invasion of hepatocarcinoma cells under starvation. Therapeutic approaches that interfere with the above mechanism may reduce the adverse side effects of anti‐angiogenesis therapy and improve the prognosis of hepatocarcinoma patients.

## CONFLICT OF INTEREST

The authors report no conflict of interest.
