# Supplementary figures and images for: Autophagy induces transforming growth factor‐β‐dependent epithelial‐mesenchymal transition in hepatocarcinoma cells through cAMP response element binding signalling
Source: J Cell Mol Med. 2018 Aug 22;22(11):5518–32. doi: 10.1111/jcmm.13825 (PMC6201351; doi:10.1111/jcmm.13825)

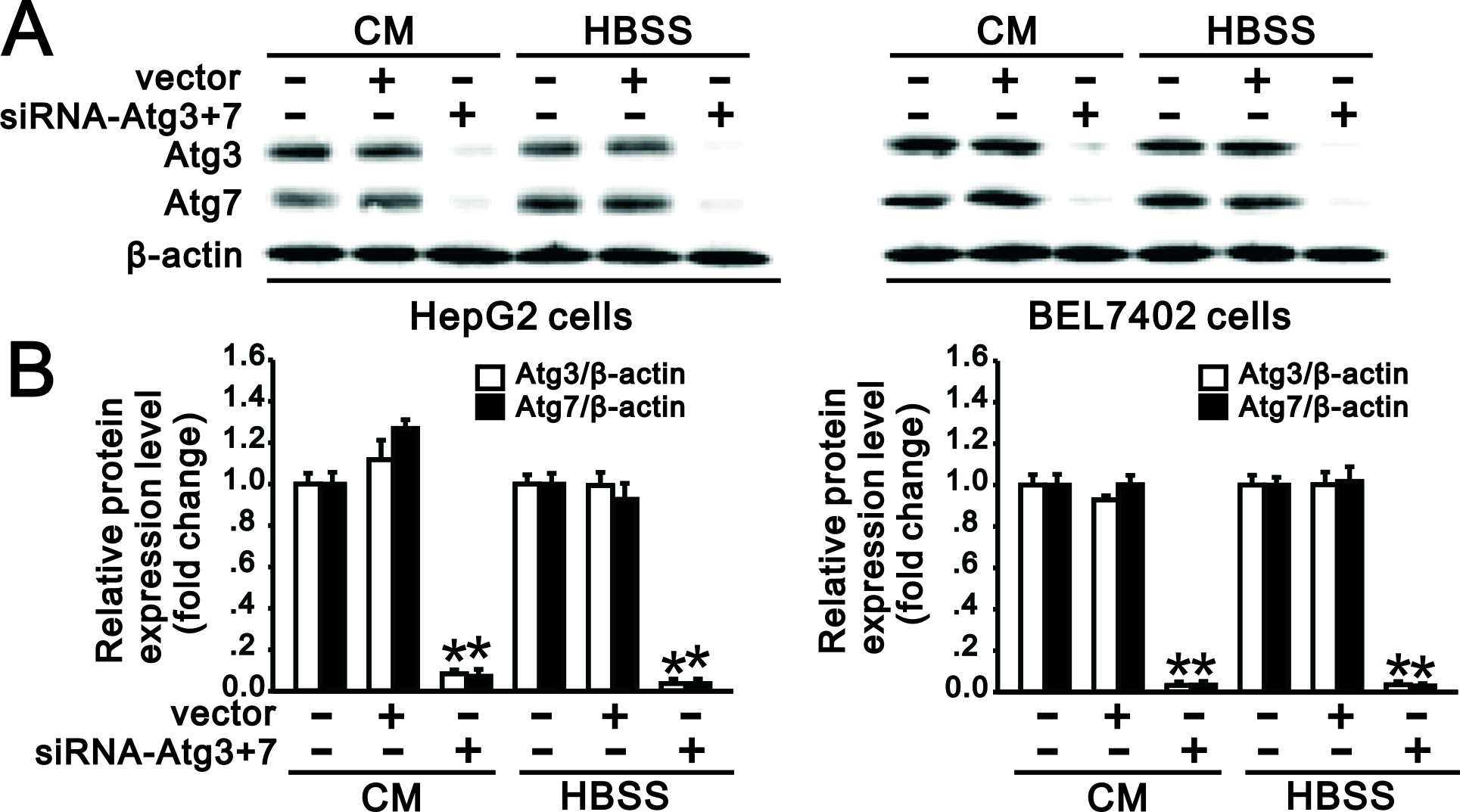

Supplement: Supplementary file 1 [file JCMM-22-5518-s001.tif]
