# Supplementary material for: Autophagy induces transforming growth factor‐β‐dependent epithelial‐mesenchymal transition in hepatocarcinoma cells through cAMP response element binding signalling
Source: J Cell Mol Med. 2018 Aug 22;22(11):5518–32. doi: 10.1111/jcmm.13825 (PMC6201351; doi:10.1111/jcmm.13825)
Supplement: Supplementary file 2 [file JCMM-22-5518-s002.docx]

**Autophagy induces transforming growth factor‐β‐dependent epithelial‐mesenchymal transition in hepatocarcinoma cells through cAMP response element binding signalling**

**Shaobo Hu^1^, Liyu Wang^1^, Xi Zhang^2^, Yongzhong Wu^3^, Jing Yang^4^*, Jun Li^5^***

**Supplemental Figure legends**

**Supplemental Figure I. *Combined* *transfection with siRNA-Atg3 and siRNA-Atg7 effectively silences Atg3 and Atg7 expression in hepatocarcinoma cell lines.***

HepG2 and BEL7402 cells were combining trensfected with siRNA-Atg3 and siRNA-Atg7. After 48 hours of transfection, the transfected cells were cultured in complete medium or in HBSS. The expression of Atg3 and Atg7 was measured by Western blot. (A) Representative Western blots and (B) densitometric analysis for Atg3 and Atg7 expression normalized to β-actin in HepG2 (left panel) and BEL7402 cells (right panel) in complete medium or in HBSS. Cells without transfection served as control. Data are representative of 3 independent experiments and shown as mean ± SEM, n=3, *P < 0.05 versus control.
